# Test-Retest Reliability and Interpretation of Common Concussion Assessment Tools: Findings from the NCAA-DoD CARE Consortium

**DOI:** 10.1007/s40279-017-0813-0

**Published:** 2017-11-14

**Authors:** Steven P. Broglio, Barry P. Katz, Shi Zhao, Michael McCrea, Thomas McAllister, CARE Consortium Investigators, April Reed Hoy, Joseph Hazzard, Louise Kelly, Justus Ortega, Nicholas Port, Margot Putukian, Dianne Langford, Darren Campbell, Gerald McGinty, Patrick O’Donnell, Steven Svoboda, John DiFiori, Christopher Giza, Holly Benjamin, Thomas Buckley, Thomas Kaminski, James Clugston, Julianne Schmidt, Luis Feigenbaum, James Eckner, Kevin Guskiewicz, Jason Mihalik, Jessica Miles, Scott Anderson, Christina Master, Anthony Kontos, Sara Chrisman, Alison Brooks, Stefan Duma, Christopher Miles, Brian Dykhuizen, Laura Lintner

**Affiliations:** 1 NeuroTrauma Research Laboratory, University of Michigan Injury Center, University of Michigan, 401 Washtenaw Ave, Ann Arbor, MI 48109 USA; 2 Department of Biostatistics, Indiana University, Indianapolis, IN USA; 3 Departments of Neurosurgery and Neurology, Medical College of Wisconsin, Milwaukee, WI USA; 4 Department of Psychiatry, Indiana University School of Medicine, Indianapolis, IN USA

## Abstract

**Background:**

Concussion diagnosis is typically made through clinical examination and supported by performance on clinical assessment tools. Performance on commonly implemented and emerging assessment tools is known to vary between administrations, in the absence of concussion.

**Objective:**

To evaluate the test-retest reliability of commonly implemented and emerging concussion assessment tools across a large nationally representative sample of student-athletes.

**Methods:**

Participants (*n* = 4874) from the Concussion Assessment, Research, and Education Consortium completed annual baseline assessments on two or three occasions. Each assessment included measures of self-reported concussion symptoms, motor control, brief and extended neurocognitive function, reaction time, oculomotor/oculovestibular function, and quality of life. Consistency between years 1 and 2 and 1 and 3 were estimated using intraclass correlation coefficients or Kappa and effect sizes (Cohen’s *d*). Clinical interpretation guidelines were also generated using confidence intervals to account for non-normally distributed data.

**Results:**

Reliability for the self-reported concussion symptoms, motor control, and brief and extended neurocognitive assessments from year 1 to 2 ranged from 0.30 to 0.72 while effect sizes ranged from 0.01 to 0.28 (i.e., small). The reliability for these same measures ranged from 0.34 to 0.66 for the year 1–3 interval with effect sizes ranging from 0.05 to 0.42 (i.e., small to less than medium). The year 1–2 reliability for the reaction time, oculomotor/oculovestibular function, and quality-of-life measures ranged from 0.28 to 0.74 with effect sizes from 0.01 to 0.38 (i.e., small to less than medium effects).

**Conclusions:**

This investigation noted less than optimal reliability for most common and emerging concussion assessment tools. Despite this finding, their use is still necessitated by the absence of a gold standard diagnostic measure, with the ultimate goal of developing more refined and sound tools for clinical use. Clinical interpretation guidelines are provided for the clinician to apply with a degree of certainty in application.

**Electronic supplementary material:**

The online version of this article (10.1007/s40279-017-0813-0) contains supplementary material, which is available to authorized users.

## Key Points

**Table:** 

Understanding normal performance variation on standard clinical assessments of concussion is vital to application and interpretation in the clinical setting.
Commonly implemented concussion assessments do not meet the necessary threshold at 1- and 2-year testing intervals.
Change scores are provided to give the clinician a degree of confidence when interpreting post-injury results.

## Introduction

Multiple organizations suggest [1, 2] or endorse [3, 4] athletes engaging in sports that carry a concussion risk undergo a baseline evaluation prior to participation, against which to measure impairments resulting from injury. The baseline assessment also permits medical professionals caring for concussed athletes to apply individualized performance metrics when determining if the athlete is concussed and/or when to allow for a return to play. Important in this process is understanding the foundational psychometric properties of the clinical measures. Test reliability, the level of stability of a test administered on more than one occasion, is one such metric that influences clinical decision making by identifying normal variation within the test vs. variation attributed to a concussion. Ideally, in the absence of injury, there should be minimal performance variation on measures that evaluate stable traits such as neurocognitive function and motor control. State and trait variance precludes perfect stability, thus it is critical to know the degree of normal variation on a measure to determine clinically meaningful performance changes that can reliability be attributed to injury. As the concussion diagnosis can only be made through a clinical examination, test reliability is of particular importance to the healthcare provider who does not know the true health status of the athlete and must rely on clinical measures to assist in the injury management process.

Within the sports medicine community, there is broad support for the inclusion of measures of neurocognitive function, motor control, and athlete-reported symptoms to be used in conjunction with the clinical examination. In addition, emerging assessments that evaluate eye tracking, vestibular-ocular function, reaction time, and quality of life are beginning to be implemented. Collectively, these measures are used to support the clinical examination for concussion [1]. Previous research has evaluated the reliability of each of these with varying results.

A wide range of reliabilities [e.g., intraclass correlation coefficients (ICCs), Pearson’s *r*, generalizability coefficient (*G*)] have been reported for computer-based neurocognitive assessments, including the Immediate Post-Concussion Assessment and Cognitive Test [ImPACT] (ICC = 0.23–0.88), Automated Neuropsychological Assessment Metrics (ICC = 0.14–0.86), and the Cogstate Computerized Cognitive Assessment Tool (CCAT, formerly named Axon: ICC = 0.45–0.90) [5]. The Standardized Assessment of Concussion (SAC), a neurocognitive screening tool, has been reported at *r* = 0.48 [6], while the Balance Error Scoring System (BESS), a measure of motor control, is reported at *G* = 0.63 among male individuals and *G* = 0.60 for female individuals when administered one time [7]. Similarly, the King-Devick test, a measure of eye tracking, has been reported at ICC = 0.95 in a collegiate athlete sample [8]. Variable test performance can be associated with a number of factors including sleep [9], testing environment [10], and the test-retest interval [5]. While useful, each of the aforementioned studies has analyzed performance from relatively small cohorts and failed to include athletes from varying sexes, a wide breadth of sports, or skill levels.

While ICCs give a measure of a specific assessment’s stability over time, interpreting performance changes relative to concussion is vital for clinical application. Reliable change indices (RCIs) place a positive and negative range around a pre-morbid score based on statistical confidence [11] and have been calculated for many of the measures noted above. In the case of concussion, worsening scores that exceed this range following a head impact are typically attributed to the concussive injury. Reliable change indices have been applied to computerized neurocognitive assessments [12], neurocognitive screening [13], motor control [7], and concussion-related symptoms[14] for clinical interpretation. While broadly applied in the past, RCIs are calculated using a bi-directional confidence interval, although only performance declines are of interest following a suspected injury. In addition, RCIs assume a normal distribution, which is not always the case with concussion-related assessments. For example, baseline symptom reports are often right skewed with a mean close to zero while SAC performance is left skewed with many individuals scoring at or near maximum performance. Therefore, the intent of this investigation is to evaluate data collected as part of a prospective investigation on the natural history of concussion from a multi-site consortium to establish the test-retest reliability and clinical interpretation ranges for a number of accepted and emerging concussion assessment measures.

## Methods

Between 2014 and 2017, the Concussion Assessment, Research, and Education (CARE) Consortium conducted a 30-site investigation on the 6-month natural history of concussion. All National Collegiate Athletic Association university student athletes and all cadets at the participating military service academies were eligible for participation and all participants provided written informed consent following protocol approval by both the institution’s local institutional review board and the US Army Human Research Protection Office. This study was completed in accordance with the Declaration of Helsinki.

The CARE methods have been described in detail elsewhere [15]. Briefly, at the time of enrollment and following consent, each participant completed a detailed demographics questionnaire and then completed a baseline assessment. The assessments were divided into mandatory (Level A measures) and optional emerging concussion measures (Level B measures) for each of the following. Level A domains (assessment name and number of sites providing data) included: neurocognitive screening (SAC, *n* = 29 sites), motor control [BESS, *n* = 29 sites], symptoms [Standardized Concussion Assessment Tool (SCAT) symptom inventory, *n* = 29 sites; Brief Symptom Inventory (BSI)-18, *n* = 29 sites], and neurocognitive function [ImPACT, *n* = 25 sites; Computerized Neurocognitive Software Vital Signs, *n* = 2 sites; CCAT, *n* = 1 site]. Level B measures included reaction time [clinical reaction time (RT_clin_), *n* = 3 sites], oculomotor/oculovestibular function [vestibular/ocular-motor screening (VOMS), *n* = 9 sites; King-Devick test, *n* = 6 sites], and quality of life [Satisfaction with Life Scale (SWLS), *n* = 11 sites]. The baseline assessment was completed annually for each year the participant was eligible for the study and prior to the competitive season. Time to complete the initial baseline assessment was 55–60 min and approximately 45 min each successive year. Each assessment is described in brief below:

### Level A Measures

- The *SAC* assesses cognitive status after acute injury. The SAC has demonstrated validity, reliability, and sensitivity to concussion [16]. The SAC contains sections on orientation, immediate memory, concentration, and delayed recall and takes 5 min to administer [6].
- The *BESS* is an postural stability measure that can be implemented on the sideline [17]. The test is administered in 5 min while the athlete completes three 20-s stance trials (i.e., double leg, single leg, tandem stance) on firm and foam surfaces.
- The *SCAT symptom inventory* is a 22-item list of symptoms commonly associated with concussions (e.g., headache, nausea, fatigue). Each athlete rates the presence/absence of the symptom on a 0–6 Likert scale, 0 indicating the symptom is not present and 6 being the most severe [18].
- The *BSI-18* is a brief symptom inventory designed with reliability in mind. The BSI-18 assessment gathers patient-reported data to help measure psychological distress in primary care settings and has been shown to be reliable and valid in a brain injury cohort. The assessment takes 4 min to complete [19].
- *ImPACT* is a 25-min test that generates composite scores quantifying performance in the domains of: attention span, working memory, sustained and selective attention time, non-verbal problem solving, and reaction time [20].
- *Computerized Neurocognitive Software Vital Signs* is a 25- to 30-min test designed to evaluate a number of cognitive domains such as verbal memory, visual memory, and executive functioning, through seven assessment modules [21].
- *Cogstate CCAT* is a 15-min test that contains four tasks asking the participant to respond to virtual playing cards to generate measures of processing speed, working memory, attention, and learning [22].

### Level B Measures

- *RT*_*clin*_ is a modified stick-drop test where the participant catches a numbered rod as quickly as possible and drop distance is converted to speed. The test has been shown to have moderate-to-high sensitivity in a concussed athletic population and takes 3 min to administer [23].
- *Vestibular Ocular Motor Screen* is a rapid evaluation of vestibular and ocular function. During the evaluation, the clinician evaluates smooth pursuits, saccades, convergence, fixating on a stationary object while moving the head side to side/up and down (vestibular ocular reflex), and standing while tracking a moving object by and turning the head and torso fully side to side (visual motion sensitivity) [24].
- The *King-Devick test* requires an athlete to read single digit numbers displayed on cards or an electronic tablet. After suspected head trauma, the athlete is given the test and, if the time needed to complete the test is longer than the baseline test time, the athlete should be removed from play [8].
- The *SWLS* is a five-item scale that assesses global life satisfaction in various age groups [25]. The SWLS suggests that it is sensitive enough to detect changes in life satisfaction throughout a clinical intervention [26].

At the time of this analysis, 23,590 student athletes and cadets had been enrolled and 8675 completed a baseline assessment on 2 consecutive years and 872 on 3 consecutive years. Throughout the duration of the study, each CARE participant participated in his/her sport or training without interference from the study team and cadets completed their normal physical and tactical training. In the event a participant sustained a diagnosed concussion, he/she was evaluated at five post-injury time points, but was removed from the data set included in this analysis (*n* = 1093). In addition, military service academy cadets that were not National Collegiate Athletic Association university-level student athletes were not included in the analysis (*n* = 2708), but will be described in a forthcoming publication. The final dataset included 4874 participants with variable completion rates for each assessment and year of the study.

### Data Analysis

Distribution metrics (e.g., mean, median, and quartiles) were first calculated. Reliability can be calculated in a number of ways (e.g., ICCs and Kappa). Test-retest reliability was estimated between years 1 and 2 and years 1 and 3 using a two-way mixed-model analysis of variance (ICC_3,1_) [27] for consistency between assessments. In place of ICCs, Kappa was used to calculate test-retest reliability for the SCAT symptom and symptom severity scores and VOMS measures. This approach was adopted owing to the skewed distributions exhibited by these scores. Before Kappa was estimated, data were categorized as 0, 1, 2, and ≥ 3 for the symptom score and VOMS measures and 0, 1, 2, 3, and ≥ 4 for severity. Intraclass correlation coefficients and Kappa are scored on a 0–1.0 scale with higher scores representing more stable performance. Interpretation of ICCs and Kappa scores vary in the literature with some suggesting that scores over 0.75 are representative of good reliability, while those less than 0.75 reflect moderate-to-poor reliability [28]. Others have suggested higher scores are needed in making decisions surrounding concussion diagnosis and management [29]. Cohen’s *d* effect sizes were also calculated to evaluate the magnitude of change between years 1 and 2 and years 1 and 3. Interpretation was based on recommendations provided by Cohen [30], whereby estimates < 0.2 are deemed small, 0.5 is a medium effect, and 0.8 is a large effect.

Intraclass correlation coefficients and other calculations were not completed when the sample was less than 100 to ensure appropriate representation of the metrics presented. This largely occurred in the year 1–3 assessments. Inferential statistics (e.g., *t* tests) were not employed to evaluate between-year differences because the large sample size would likely yield statistical significance in the presence of clinically meaningless changes.

Last, to provide clinical interpretation guidelines that did not assume normally distributed data, we applied nonparametric confidence intervals based on the observed distributions to estimate the degree of certainty of change on each assessment rather than estimating the percentiles (i.e., RCIs) of the distribution under the assumption of normality. This method is more robust when normality cannot be assumed because, for large sample sizes, the empirical distribution converges to the true distribution by the strong law of large numbers [31]. All calculations were completed using R Version 3.4.0 statistical software package (Vienna, Austria).

## Results

Data analysis included 4874 (41.09% female) university-level student athletes from 29 National Collegiate Athletic Association institutions. Participant demographics at the time of the initial baseline assessment were: 19.2 ± 1.2 years (age), 178.3 ± 10.96 cm (height), 78.9 ± 1 9.1 kg (weight), and 0.4 ± 0.8 concussions reported prior to enrollment. The mean time between the first and second assessment was 316.1 ± 83.4 days and the first and third assessments were separated by 627.5 ± 99.8 days.

Distribution metrics and reliability analysis results for the Level A SAC, BESS, SCAT (symptom total and severity), and BSI-18 are presented in Table 1. Level A neurocognitive measures are presented in Tables 2 (ImPACT and CCAT) and Table 3 (Computerized Neurocognitive Software Vital Signs). Distribution metrics and reliability analysis results for the optional Level B Clinical Reaction Time, VOMS, King-Devick test, and SWLS are presented in Table 4. Baseline performance metrics for the entire cohort and several sub-cohorts have been presented elsewhere [32] and are consistent with the data presented here. Reliability for the Level A assessments from the year 1–2 assessments ranged from 0.30 to 0.72 and the year 1–3 assessments ranged from 0.34 to 0.66 (Table 1). Overall, the reliability analysis indicated slightly lower consistency for the year 1–3 assessment compared with the year 1–2. The year 1–2 reliability for the Level B measures ranged from 0.28 to 0.74 (Table 4), but only one measure (SWLS) had a large enough sample to generate reliability for years 1–3. Overall, the ImPACT Visual Motor Speed and King-Devick test were the only evaluations that neared 0.75, suggesting good reliability for years 1–2 [28].

**Table 1:** Measures of central tendency, reliability, and effect sizes for Level A clinical concussion measures

SAC	*n*	Mean (SD)	Median	1st quartile	3rd quartile	ICC (lower, upper bound)	Cohen’s *d*
Year 1	3208	27.25 (2.03)	27	26	29	0.39 (0.36–0.42)	0.07
Year 2		27.39 (1.91)	28	26	29		
Year 1	372	27.25 (1.99)	28	26	29	0.34 (0.24–0.42)	0.42
Year 3		28.01 (1.65)	28	27	29		

BESS	*n*	Mean (SD)	Median	1st quartile	3rd quartile	ICC (lower, upper bound)	Cohen’s *d*
Year 1	2894	13.15 (6.06)	12	9	17	0.41 (0.38–0.44)	0.28
Year 2		11.50 (5.57)	11	7	14		
Year 1	323	11.95 (5.58)	11	8	15	0.42 (0.32–0.50)	0.24
Year 3		10.65 (5.46)	10	7	14		

SCAT: total symptoms	*n*	Mean (SD)	Median	1st quartile	3rd quartile	Kappa	Cohen’s *d*
Year 1	4360	0.79 (0.77)	1	0	1	0.40	0.10
Year 2		0.71 (0.74)	1	0	1		
Year 1	581	0.72 (0.74)	1	0	1	0.42	0.05
Year 3		0.72 (0.78)	1	0	1		

SCAT: symptom severity	*n*	Mean (SD)	Median	1st quartile	3rd quartile	Kappa	Cohen’s *d*
Year 1	4360	0.98 (1.04)	1	0	2	0.41	0.11
Year 2		0.88 (0.98)	1	0	1		
Year 1	581	0.86 (0.96)	1	0	1	0.41	0.05
Year 3		0.87 (1.00)	1	0	1		

BSI-18	*n*	Mean (SD)	Median	1st quartile	3rd quartile	ICC (lower, upper bound)	Cohen’s *d*
Year 1	4328	2.67 (4.80)	1	0	3	0.38 (0.35–0.40)	0.13
Year 2		2.09 (4.50)	0	0	2		
Year 1	551	2.52 (4.05)	1	0	3	0.51 (0.44–0.57)	0.19
Year 3		1.80 (3.73)	0	0	2		

*BESS* balance error scoring system, *BSI* brief symptom inventory, *ICC* intraclass correlation coefficient, *SAC* standardized assessment of concussion, *SCAT* standardized concussion assessment tool, *SD* standard deviation

**Table 2:** Measures of central tendency, reliability, and effect sizes for Level A neurocognitive measures

ImPACT verbal memory	*n*	Mean (SD)	Median	1st quartile	3rd quartile	ICC (lower, upper bound)	Cohen’s *d*
Year 1	3154	87.90 (10.36)	90	81	96	0.50 (0.48–0.53)	0.05
Year 2		88.40 (10.52)	91	82	97		
Year 1	505	87.61 (10.78)	90	81	96	0.47 (0.40–0.53)	0.23
Year 3		90.00 (9.93)	93	84	99		

ImPACT visual memory	*n*	Mean (SD)	Median	1st quartile	3rd quartile	ICC (lower, upper bound)	Cohen’s *d*
Year 1	3154	78.24 (13.31)	80	70	89	0.58 (0.55–0.60)	0.11
Year 2		79.65 (13.25)	81	72	90		
Year 1	505	78.04 (13.36)	80	70	89	0.47 (0.40–0.54)	0.22
Year 3		80.86 (12.84)	82	73	91		

ImPACT visual motor speed	*n*	Mean (SD)	Median	1st quartile	3rd quartile	ICC (lower, upper bound)	Cohen’s *d*
Year 1	3154	41.78 (6.47)	42.12	37.17	47.02	0.72 (0.70–0.74)	0.13
Year 2		42.64 (6.42)	43.29	38.2	47.75		
Year 1	505	42.11 (6.41)	42.4	37.6	47.08	0.66 (0.61–0.71)	0.22
Year 3		43.47 (5.98)	44.03	39.47	48.22		

ImPACT reaction time	*n*	Mean (SD)	Median	1st quartile	3rd quartile	ICC (lower, upper bound)	Cohen’s d
Year 1	3149	0.5867 (0.09)	0.57	0.53	0.63	0.47 (0.44–0.50)	0.05
Year 2		0.5828 (0.08)	0.57	0.53	0.62		
Year 1	503	0.598 (0.11)	0.57	0.53	0.64	0.34 (0.26–0.42)	0.14
Year 3		0.585 (0.08)	0.58	0.53	0.63		

CCAT composite processing speed^a^	*n*	Mean (SD)	Median	1st quartile	3rd quartile	ICC (lower, upper bound)	Cohen’s *d*
Year 1	447	102.76 (6.11)	103.8	100.1	106.5	0.49 (0.41–0.55)	0.09
Year 2		103.31 (5.86)	104.2	100.9	107		

CCAT composite attention^a^	*n*	Mean (SD)	Median	1st quartile	3rd quartile	ICC (lower, upper bound)	Cohen’s d
Year 1	448	106.39 (4.71)	107	103.8	109.82	0.56 (0.49–0.62)	0.01
Year 2		106.36 (5.24)	106.9	103.3	109.82		

CCAT composite learning^a^	*n*	Mean (SD)	Median	1st quartile	3rd quartile	ICC (lower, upper bound)	Cohen’s *d*
Year 1	446	104.64 (10.25)	103.7	98.2	111.2	0.54 (0.47–0.6)	0.28
Year 2		107.46 (10.12)	107.3	100.73	115.3		

CCAT working memory speed: speed^a^	*n*	Mean (SD)	Median	1st quartile	3rd quartile	ICC (lower, upper bound)	Cohen’s *d*
Year 1	448	103.52 (6.15)	104	99.38	107.8	0.59 (0.53–0.65)	0.15
Year 2		104.47 (6.1)	104.5	100.57	108.5		

*CCAT* computerized concussion assessment tool, *ICC* intraclass correlation coefficient, *ImPACT* immediate post-concussion assessment and cognitive test, *SD* standard deviation

^a^Indicates insufficient sample size to complete the year 1–3 estimates

**Table 3:** Measures of central tendency, reliability, and effect sizes for Level A neurocognitive measure

CNS neurocognition index^a^	*n*	Mean (SD)	Median	1st quartile	3rd quartile	ICC (lower, upper bound)	Cohen’s *d*
Year 1	238	98.36 (10.82)	98.5	93.25	105	0.33 (0.21–0.44)	0.01
Year 2		98.21 (12.71)	100	93	106		

CNS composite memory standard score^a^	*n*	Mean (SD)	Median	1st quartile	3rd quartile	ICC (lower, upper bound)	Cohen’s *d*
Year 1	238	95.72 (16.3)	96	86	106.75	0.43 (0.32–0.53)	0.07
Year 2		96.89 (17.28)	97	86	108		

CNS verbal memory standard score^a^	*n*	Mean (SD)	Median	1st quartile	3rd quartile	ICC (lower, upper bound)	Cohen’s *d*
Year 1	238	94.68 (18.1)	97	83	108	0.41 (0.29–0.51)	0.02
Year 2		95.07 (20.17)	97	86	109		

CNS visual memory standard score^a^	*n*	Mean (SD)	Median	1st quartile	3rd quartile	ICC (lower, upper bound)	Cohen’s *d*
Year 1	238	98.2 (15.1)	98	90	110	0.31 (0.19–0.42)	0.06
Year 2		99.05 (15.46)	101	91	110		

CNS psychomotor speed standard score^a^	*n*	Mean (SD)	Median	1st quartile	3rd quartile	ICC (lower, upper bound)	Cohen’s *d*
Year 1	239	105.3 (13.54)	106	98	113.5	0.58 (0.49–0.66)	0.06
Year 2		104.5 (13.53)	105	95.5	113		

CNS reaction time standard score^a^	*n*	Mean (SD)	Median	1st quartile	3rd quartile	ICC (lower, upper bound)	Cohen’s *d*
Year 1	240	97 (16.31)	99	89	108	0.53 (0.43–0.62)	0.02
Year 2		96.77 (14.17)	98	88	107		

CNS complex attention standard score^a^	*n*	Mean (SD)	Median	1st quartile	3rd quartile	ICC (lower, upper bound)	Cohen’s *d*
Year 1	237	98.14 (14.64)	101	89	110	0.49 (0.39–0.58)	0.05
Year 2		97.39 (16.18)	101	92	107		

CNS cognitive flexibility standard score^a^	*n*	Mean (SD)	Median	1st quartile	3rd quartile	ICC (lower, upper bound)	Cohen’s *d*
Year 1	240	97.51 (14.15)	98	92	107	0.40 (0.28–0.5)	0.2
Year 2		100.2 (13.22)	102	92	109		

CNS processing speed standard score^a^	*n*	Mean (SD)	Median	1st quartile	3rd quartile	ICC (lower, upper bound)	Cohen’s *d*
Year 1	240	102.38 (14.8)	101	94	110.25	0.61 (0.52–0.68)	0.04
Year 2		103 (16.16)	102	93	112		

CNS executive function standard score^a^	*n*	Mean (SD)	Median	1st quartile	3rd quartile	ICC (lower, upper bound)	Cohen’s *d*
Year 1	240	98.57 (13.68)	99	93	107	0.44 (0.33–0.54)	0.26
Year 2		101.88 (11.36)	102.5	94	110		

CNS simple attention percentile	*n*	Mean (SD)	Median	1st quartile	3rd quartile	ICC (lower, upper bound)	Cohen’s *d*
Year 1	267	46.63 (26.93)	50	23	70	0.30 (0.19–0.41)	0.13
Year 2		43.29 (26.55)	40	16	70		

CNS motor speed standard score^a^	*n*	Mean (SD)	Median	1st quartile	3rd quartile	ICC (lower, upper bound)	Cohen’s *d*
Year 1	239	105.13 (12.79)	105	99	112	0.53 (0.44–0.62)	0.11
Year 2		103.87 (10.95)	103	97	110		

*CNS* computerized neurocognitive software, *ICC* intraclass correlation coefficient, *SD* standard deviation

^a^Indicates insufficient sample size to complete the year 1–3 estimates

**Table 4:** Measures of central tendency, reliability, and effect sizes for Level B concussion assessments

Clinical reaction time^a^	*n*	Mean (SD)	Median	1st quartile	3rd quartile	ICC (lower, upper bound)	Cohen’s *d*
Year 1	261	198.26 (22.57)	197	184	213	0.32 (0.21–0.43)	0.34
Year 2		190.61 (22.63)	190	176	206		

VOMS smooth pursuit^a^	*n*	Mean (SD)	Median	1st quartile	3rd quartile	Kappa	Cohen’s *d*
Year 1	525	0.34 (1.07)	0	0	0	0.3	0.01
Year 2		0.35 (1.04)	0	0	0		

VOMS horizontal saccades^a^	*n*	Mean (SD)	Median	1st quartile	3rd quartile	Kappa	Cohen’s *d*
Year 1	525	0.39 (1.12)	0	0	0	0.29	0.01
Year 2		0.4 (1.17)	0	0	0		

VOMS vertical saccades^a^	*n*	Mean (SD)	Median	1st quartile	3rd quartile	Kappa	Cohen’s *d*
Year 1	524	0.4 (1.17)	0	0	0	0.28	0.02
Year 2		0.42 (1.18)	0	0	0		

VOMS near point convergence symptoms^a^	*n*	Mean (SD)	Median	1st quartile	3rd quartile	Kappa	Cohen’s *d*
Year 1	474	0.45 (1.29)	0	0	0	0.36	0.01
Year 2		0.44 (1.28)	0	0	0		

VOMS near point convergence distance^a^	*n*	Mean (SD)	Median	1st quartile	3rd quartile	Kappa	Cohen’s *d*
Year 1	521	2.16 (2.76)	1	0	3	0.51	0.01
Year 2		2.14 (3.15)	1	0	3		

VOMS VOR horizontal^a^	*n*	Mean (SD)	Median	1st quartile	3rd quartile	Kappa	Cohen’s *d*
Year 1	525	0.56 (1.48)	0	0	0	0.38	0.19
Year 2		0.59 (1.44)	0	0	0		

VOMS VOR vertical^a^	*n*	Mean (SD)	Median	1st quartile	3rd quartile	Kappa	Cohen’s *d*
Year 1	525	0.52 (1.48)	0	0	0	0.38	0.02
Year 2		0.55 (1.43)	0	0	0		

VOMS visual motion sensitivity^a^	*n*	Mean (SD)	Median	1st quartile	3rd quartile	Kappa	Cohen’s *d*
Year 1	521	0.54 (1.56)	0	0	0	0.35	0.01
Year 2		0.52 (1.43)	0	0	0		

King-Devick	*n*	Mean (SD)	Median	1st quartile	3rd quartile	ICC (lower, upper bound)	Cohen’s *d*
Year 1	755	43.78 (7.83)	42.85	38.26	48.44	0.74 (0.70–0.77)	0.38
Year 2		40.90 (7.40)	40.06	35.68	45.49		

Satisfaction with life scale	*n*	Mean (SD)	Median	1st quartile	3rd quartile	ICC (lower, upper bound)	Cohen’s *d*
Year 1	966	29.20 (4.86)	30	27	33	0.49 (0.44–0.54)	0.18
Year 2		28.32 (4.92)	29	26	31		
Year 1	166	29.50 (4.42)	30	28	32	0.42 (0.28–0.53)	0.21
Year 3		28.51 (4.80)	29	27	31		

*ICC* intraclass correlation coefficient, *VOMS* vestibular/ocular-motor screening, *VOR* vestibular ocular reflex*, SD* standard deviation

^a^Indicates insufficient sample size to complete the year 1–3 estimates

Cohen’s *d* calculations for the Level A measures are presented in Tables 1, 2, 3. Across all Level A measures, the year 1–2 assessment yielded effect sizes ranging from 0.01 to 0.28 (i.e., small) and the effect sizes for year 1–3 were 0.05–42 (i.e., small to less than medium). Effect sizes for the Level B measures are presented in Table 4. Across all Level B measures, the year 1–2 assessments yielded effect sizes ranging from 0.01 to 0.38 (i.e., small to less than medium effects). The SWLS was again the only Level B measure that had a large enough sample to generate year 1–3 effect sizes.

The change scores for each assessment, through a range of confidence intervals, were calculated and presented in Table 5 (Level A) and Table 6 (Level B). These estimates provide a degree of certainty to the practitioner when interpreting change in performance following a suspected concussion. Last, these same analyses have been completed individually for male and female participants and are presented in the Electronic Supplementary Material.

**Table 5:** Confidence ranks by change score for Level A concussion measures

	75%	87.5%	90%	92.5%	95%	97.5%	99%
SAC							
Years 1–2	− 1	− 2	− 2	− 3	− 3	− 4	− 5
Years 1–3	− 1	− 1	− 2	− 2	− 2.5	− 3	− 4.3
BESS							
Years 1–2	2	5	6	7	9	11.7	14
Years 1–3	2	4	5	8	9	13	14
SCAT total							
Years 1–2	1	3	3	4	5	7	10
Years 1–3	1	3	4	5	6	8	10.4
SCAT severity							
Years 1–2	1	4	6	7	9	15	24
Years 1–3	2	6	7	8	10	15	27.6
BSI-18							
Years 1–2	0	2	3	4	6	10	15.7
Years 1–3	0	2	2	3	4.5	7	10
ImPACT verbal memory							
Years 1–2	− 5	− 10	− 12	− 14	− 17	− 21	− 27
Years 1–3	− 3	− 9	− 9.6	− 12	− 14	− 18.4	− 23
ImPACT visual memory							
Years 1–2	− 6	− 12	− 14	− 16	− 18	− 23	− 28
Years 1–3	− 6	− 12	− 13.6	− 16	− 18	− 23.8	− 28
ImPACT visual motor speed							
Years 1–2	− 2.1	− 4.1	− 4.8	− 5.8	− 6.8	− 8.5	− 11.2
Years 1–3	− 1.9	− 3.9	− 4.4	− 5.1	− 5.9	− 7.4	− 9.1
ImPACT reaction time							
Years 1–2	0.04	0.07	0.08	0.09	0.12	0.15	0.22
Years 1–3	0.04	0.07	0.08	0.1	0.12	0.15	0.21
CCAT composite processing speed^a^							
Years 1–2	− 2.4	− 5.4	− 6.5	− 7.9	− 9.1	− 12.4	− 15.4
CCAT composite attention^a^							
Years 1–2	− 2.7	− 4.7	− 5	− 5.5	− 6.9	− 9.6	− 14.7
CCAT composite learning^a^							
Years 1–2	− 3.15	− 7.6	− 9.3	− 11.2	− 14.3	− 16.9	− 21.1
CCAT working memory speed: speed^a^							
Years 1–2	− 2.5	− 5.2	− 6.1	− 7.3	− 8.4	− 10.4	− 13.1
CNS neurocognition index^a^							
Years 1–2	− 5	− 9	− 10	− 11	− 13.2	− 17	− 58.2
CNS composite memory standard score^a^							
Years 1–2	− 10	− 18	− 19.6	− 24	− 27.2	− 35.2	− 40.9
CNS verbal memory standard score^a^							
Years 1–2	− 11	− 20.4	− 25.3	− 27	− 31.2	− 38.2	− 49.4
CNS visual memory standard score^a^							
Years 1–2	− 10.8	− 19.4	− 22	− 25.2	− 28.5	− 34.3	− 40.9
CNS psychomotor speed standard score^a^							
Years 1–2	− 8	− 14	− 15	− 16	− 18.1	− 21	− 29
CNS reaction time standard score^a^							
Years 1–2	− 8	− 15	− 16	− 18	− 22	− 28	− 31.6
CNS complex attention standard score^a^							
Years 1–2	− 9	− 16	− 19.4	− 21	− 25	− 32.1	− 42.8
CNS cognitive flexibility standard score^a^							
Years 1–2	− 6	− 11	− 13	− 14.1	− 17.1	− 22.1	− 37.1
CNS processing speed standard score^a^							
Years 1–2	− 7	− 12	− 15.1	− 17	− 21	− 29.1	− 37.8
CNS executive function standard score^a^							
Years 1–2	− 5	− 9	− 11	− 12	− 15	− 18	− 22.2
CNS simple attention percentile							
Years 1–2	− 29	− 39	-41	− 49	− 56	− 73	− 78
CNS motor speed standard score^a^							
Years 1–2	− 7	− 11	− 13	− 14.2	− 18.1	− 23.1	− 29.2

*BESS* balance error scoring system, *BSI* brief symptom inventory, *CCAT* computerized concussion assessment tool, *CNS* computerized neurocognitive software, *ImPACT* immediate post-concussion assessment and cognitive test, *SAC* standardized assessment of concussion, *SCAT* standardized concussion assessment tool

^a^Indicates insufficient sample size to complete the year 1–3 estimates

**Table 6:** Confidence ranks by change score for Level B concussion measures

	75%	87.50%	90%	92.50%	95%	97.50%	99%
Clinical reaction time^a^							
Years 1–2	10	22	29	31	38	45	53
VOMS smooth pursuit^a^							
Years 1–2	0	1	1	1	2	3	4
VOMS horizontal saccades^a^							
Years 1–2	0	1	1	1	2	3	4.8
VOMS vertical saccades^a^							
Years 1–2	0	1	1	1	2	3	4
VOMS near point convergence symptoms^a^							
Years 1–2	0	1	1	1	2	3.2	4.3
VOMS near point convergence distance^a^							
Years 1–2	1	2	3	3	4	6	9.8
VOMS VOR horizontal^a^							
Years 1–2	0	1	1	2	2	3.9	5
VOMS VOR vertical^a^							
Years 1–2	0	1	1	2	2	3.9	5
VOMS visual motion sensitivity^a^							
Years 1–2	0	1	1	2	2	4	4
King-Devick total time^a^							
Years 1–2	0.04	2.3	3.2	4	5.7	7.5	8.9
Satisfaction with life scale							
Years 1–2	1	3	4	4	5	8	12
Years 1–3	1	3	4	5	5	7	9

*VOMS* vestibular/ocular-motor screening, *VOR* vestibular ocular reflex

^a^Indicates insufficient sample size to complete the year 1–3 estimates

## Discussion

This investigation sought to establish the test-retest reliability and interpretation guidance for a number of widely used (Level A) and emerging (Level B) sport concussion assessments. The 1- and 2-year test intervals were selected as it is common practice for clinical personnel to evaluate their student athletes annually or once every 2 years during a collegiate career. Overall, our analysis indicates that both the commonly accepted and emerging assessments demonstrated less than optimal reliability for clinical utility [29]. Our findings are consistent with previous reports for the SAC [14], SCAT symptom total and severity [33], BSI-18 [34], and computer-based neurocognitive assessments [35–37], although the test-retest interval was shorter than implemented here. However, they are lower than previously reported for the BESS [14], RTclin [38], King-Devick [8], and VOMS [39]. Reliability analysis of the SWLS has not been completed previously in a similar cohort.

Effect sizes (i.e., Cohen’s *d*) were implemented as another measure evaluating the change between test administrations, which ranged from small to less than medium (Tables 1, 2, 3, 4). Tests demonstrating small to no effect size have substantial overlap in test performance. Indeed, small effect sizes (*d* < 0.2) represent a 92% overlap between assessments, while a medium effect size (*d* = 0.5) represents an 80% overlap. As noted in Tables 1, 2, 3, 4 and despite the lower than optimal reliability scores, the limited range of effect sizes suggests a substantial overlap between the first- and second-year or first- and third-year evaluations. This is slightly counter to the calculated ICC values, but highlights the potential that tightly clustered values may have skewed the ICCs downward (see below). In addition, the change in scores, with the exception of the SWLS, all demonstrated improvement on the assessments, suggesting a small-to-medium learning effect from years 1 to 2 and 1 to 3.

The differences between our findings and those reported previously may be explained in part by the large, diverse, and nationally representative cohort implemented here, which yielded slightly differing performances on the chosen assessments [32]. For example, performance on the SAC is consistent with previous findings of a collegiate cohort [16], but our sample was slower on the clinical reaction time by 5–15 ms [40] and the King-Devick test by 2–5 s, although the administration modality may have influenced performance on the latter [41]. Conversely, our sample performed better on the BESS test by approximately one error [16] and reported a 1.5 point lower symptom total and 0.5 point symptom severity score [42]. In addition, when examining reliability across the testing interval, assessment reliability was approximately the same between the year 1–2 and 1–3 administrations, although stability of individual tests fluctuated (Tables 1, 2, 3, 4). However, other studies have shown higher reliabilities are associated with shortening the testing interval [5]. The high degree of overlap between the year 1 and 2 and 1 and 3 performances would therefore suggest that every year or every other year, repeat baseline testing during the collegiate career may not be advantageous to concussion management, particularly when the cost/benefit trade-off of annual testing is considered. However, baseline testing should continue to be implemented, but a single administration at the time of college enrollment may suffice across the academic career. The assessment of the sensitivity and specificity of these measures alone or in combination using variable baseline assessment intervals is needed to confirm this recommendation.

Importantly, the measures included in this investigation have previously been shown to be the most sensitive to change following concussion [43, 44], but are largely non-stable cognitive assessments affected by many factors in non-injured individuals. Among other conditions common to collegiate athletes, lack of sleep [45], anxiety [46], psychiatric disorders [47], or apathy from repeat baseline testing can all influence test performance to a degree equivalent to a concussive injury. As such, it is unlikely that any measure will achieve perfect or near-perfect stability when the underlying function is not constant over time with variability in both state and trait function. Therefore, establishing a range of normal variation on these measures allows the clinician to consistently identify a change from baseline performance that is outside normal variation as clinically meaningful and the result of injury.

To assist in the interpretation of these concussion assessment tools in the clinical setting, we calculated change scores with an associated level of certainty (i.e., confidence intervals; Tables 5, 6). The scores within each interval offer an associated level of confidence the clinician can hold when the post-injury score meets or exceeds that value. For example, if a SAC score declines by five points, the clinician can have 99% confidence that the change is related to something (e.g., concussion) other than normal test-retest variability. Similarly, a six-point increase in the SCAT symptom severity would carry 90% confidence. This approach differs from previous works implementing differing statistical methods (e.g., reliable change index) that identified firm thresholds that placed clinically meaningful change into a yes/no dichotomy. However, the confidence continuum is in line with emerging thought that concussion is not immediately present at the time of impact but can evolve over time, leading to variable levels of diagnostic certainty [48]. Indeed, our approach to concussion assessment interpretation could be overlaid with clinical features (e.g., signs and symptoms) of the injury to establish diagnostic certainty in a way that tests exceeding 90% would represent ‘definite’ concussions, those between 50 and 90% are ‘probable’ concussions, and those < 50% are ‘possible’ concussions. To verify this approach and refine the confidence ranges, future works should apply the scores from the assessments to both concussed and control athletes both individually and in unison to establish their sensitivity and specificity.

Despite lower than acceptable reliability on the majority of the instruments evaluated here, these assessment tools are endorsed by a number of organizations [18, 49, 50] and are broadly implemented in the clinical setting. The reliance on consensus and clinical experience to implement these measures is at odds with the reliability metrics presented herein, but ICCs can be artificially lowered when the variability among participants is small. That is, when scores are tightly clustered (see the quartile range in Tables 1, 2, 3, 4) the ICC calculation can fall to or below zero (i.e., negative value) or exceed 1.0. This did not occur in our estimates, indicating all of our scores are valid [28]. Our large sample size would not have affected our estimates, rather it provided a stable confidence interval [51]. Despite the limitation in calculating the ICC, this method is still preferred over a Pearson *r*, which evaluates the relationship between measurements, making it unsuitable for this application [52]. Ultimately, while none of these measures individually meet the reliability standards set for clinical utility, there is evidence that combining them in a multifaceted assessment model provides a high level of sensitivity by comparing baseline performance to post-concussion changes in cognitive functioning [43, 44, 53]. Future works should evaluate the post-concussion sensitivity and specificity of the measures included herein both alone and in combination to mitigate concerns surrounding less than optimal reliability.

This investigation is not without limitation. Perhaps most notable is the assumption that all student athletes provided an honest effort during the test administrations. The computer-based neurocognitive assessments contain embedded validity checks and if a participant was flagged as invalid, he/she was asked to repeat the exam an additional time. A second invalid test did not mandate an additional assessment, but this was a small number relative to the entire sample (*n* = 21). The other assessments do not have similar validity checks. This is of particular importance as some athletes may intentionally underperform on a baseline evaluation in an effort to hide poor post-concussion performance at a later date [54] or the athletes may become apathetic with multiple years of testing. In addition, our testing intervals (1 and 2 years) are considerably longer than the typical time from a baseline assessment to injury [55], potentially resulting in lower reliability values [5]. We also note that our participants demonstrated improvement on some measures, suggesting a learning effect. Clinicians may consider administering practice tests to reduce these effects, although we did not evaluate for this; nor did we ask if the athlete completed these same measures in the past (e.g., high school athletics). Last, these findings may not apply to younger athletes who are continuing to undergo significant brain growth and development [56–59] that would warrant annual baseline assessments [49] or to professional athletes who have likely completed full brain maturation.

While brain development can vary widely between individuals, it is well accepted that the adolescent brain undergoes a period of rapid gray matter production during the teenage years, particularly in the pre-frontal cortex. The pre-frontal cortex reaches a peak volume around the age of 12 years, but it is among the last regions of the brain to achieve full functional maturation in the mid-20 s [56]. The transition from peak volume to peak efficiency is a result of gray matter pruning that streamlines the most often used cortical pathways [56]. The influence the changing cerebral structure has on cognitive performance has been demonstrated with cross-sectional work showing an overall age-related difference in test performance with older athletes performing better than younger athletes [60]. As such, the prospect of a single concussion assessment baseline in an adolescent population is likely not prudent.

## Conclusion

In the final analysis, this investigation provides a foundational psychometric evaluation of commonly implemented concussion assessment tools among collegiate athletes. None of these measures met or exceeded the accepted threshold for clinical utility, but ongoing revision and refinement are recommended over abandoning their use. Most measures fell well below levels of clinical utility, although the King-Devick test (years 1–2) and ImPACT-Visual Motor Speed (years 1–2) approached an acceptable level. Despite these findings, the overlap between assessment times was substantial for all measures, despite fluidity of their underlying constructs. As such, the annual baselines captured here likely represent state function of overt traits that will continue to vary with more testing. Therefore, among collegiate athletes, baseline assessments beyond the initial evaluation will likely not equate to better injury management. This raises the question about the value of annual baseline assessments in collegiate athletes, as repeat testing may not provide any additional clinical information beyond the initial evaluation. Despite the findings presented herein, the sensitivity of these measures alone or in combination must be completed before altering the existing standard of care.

## Electronic supplementary material

Below is the link to the electronic supplementary material.

Supplementary material 1 (XLSX 26 kb)
