# Correction to: Test-Retest Reliability and Interpretation of Common Concussion Assessment Tools: Findings from the NCAA-DoD CARE Consortium

**DOI:** 10.1007/s40279-018-0906-4

**Published:** 2018-03-26

**Authors:** Steven P. Broglio, Barry P. Katz, Shi Zhao, Michael McCrea, Thomas McAllister, CARE Consortium Investigators, April Reed Hoy, Joseph Hazzard, Louise Kelly, Justus Ortega, Nicholas Port, Margot Putukian, Dianne Langford, Darren Campbell, Gerald McGinty, Patrick O’Donnell, Steven Svoboda, John DiFiori, Christopher Giza, Holly Benjamin, Thomas Buckley, Thomas Kaminski, James Clugston, Julianne Schmidt, Luis Feigenbaum, James Eckner, Kevin Guskiewicz, Jason Mihalik, Jessica Miles, Scott Anderson, Christina Master, Anthony Kontos, Sara Chrisman, Alison Brooks, Stefan Duma, Christopher Miles, Brian Dykhuizen, Laura Lintner

**Affiliations:** 1 NeuroTrauma Research Laboratory, University of Michigan Injury Center, University of Michigan, 401 Washtenaw Ave, Ann Arbor, MI 48109 USA; 2 Department of Biostatistics, Indiana University, Indianapolis, IN USA; 3 Departments of Neurosurgery and Neurology, Medical College of Wisconsin, Milwaukee, WI USA; 4 Department of Psychiatry, Indiana University School of Medicine, Indianapolis, IN USA

## Correction to: Sports Med 10.1007/s40279-017-0813-0

The article Test-Retest Reliability and Interpretation of Common Concussion Assessment Tools: Findings from the NCAA-DoD CARE Consortium, written by Steven P. Broglio, Barry P. Katz, Shi Zhao, Michael McCrea, Thomas McAllister and CARE Consortium Investigators was originally published electronically on the publisher’s internet portal (currently SpringerLink) on 14 November 2017 without open access. With the author(s)’ decision to opt for Open Choice the copyright of the article changed on 22 March 2018 to © The Author(s) 2018 and the article is forthwith distributed under the terms of the Creative Commons Attribution 4.0 International License (http://creativecommons.org/licenses/by/4.0/), which permits use, duplication, adaptation, distribution and reproduction in any medium or format, as long as you give appropriate credit to the original author(s) and the source, provide a link to the Creative Commons license and indicate if changes were made.

The original article has been corrected.

